# Items

**Published:** 1900-03

**Authors:** 


### ITEMS.

THE SANDER PRIZE.—THE following rules for competition for the gold medal of the value of $100, dedicated by Mr. Enno Sander, of St. Louis, for the best paper on Military surgery, presented at the annual meeting of the Association of Military Surgeons of the United States, are promulgated:

1. Competition to be open to all members of the association.

2. Each competitor shall send three copies of his essay, in a sealed envelope, to the secretary, Lieut-Col. Charles Adams, Central Music Hall, Chicago, Ill., on or before April 15, 1900. The essay must be strictly anonymous, but the author shall adopt some *nom de plume* and sign the same to the essay, followed by a figure corresponding with the number of pages of the manuscript. A sealed envelope bearing the *nom de plume* on the outside, and enclosing full name and address shall accompany the essay. This envelope to be opened in the meeting of the association after the decision of the committee on the prize essay has been received.

3. The committee will designate the essay worthy of the prize, and also in their order of merit those deserving of honorable mention. Should the committee deem proper, it may recommend neither prize nor honorable mention.

4. The successful paper shall be published in the Transactions of the Association.

The committee is composed as follows: Col. N. Senn, Surgeon-General, I. N. G.; Major A. C. Girard, Surgeon, U. S. A.; Captain George W. Woods, Medical Director, U. S. N.

---

An excursion to the Paris Exposition and the International Medical Congress for physicians and their families is in contemplation under charge of Mr. Chas. Wood Fassett, of St. Joseph, Mo. It appears upon information from headquarters, recently given out, that the boat is nearly filled, there being but a few more choice berths unsold. As soon as the last stateroom is sold, the books will be closed. Steamer rates have advanced on all other lines. A deposit of $25.00 should be sent at once. Those who find they will be unable to undertake the trip will have no difficulty in disposing of their berths, and money will be refunded.

Physicians desiring to prolong their stay in Paris and London, or to take side trips, may secure passage immediately after the meeting of the American Medical Association, at Atlantic City, on the American Liner, "St. Louis," which sails June 13th, joining the main party either in London or Paris. A few select berths are unsold but application must be made at once to secure them. This trip, thirty-eight days, all expenses, can be made for $285.00. Return tickets good for one year. International Medical Congress opens August 9th, in Paris.

Remittances may be made direct to steamship agent, Mr. F. C. Clark, 111 Broadway, New York City, stating which party you wish to join, or to Charles Wood Fassett, in care Tootle, Lemon & Co., Bankers, St. Joseph, Mo., who will receipt for all remittances, and forward same to Mr. Clark.

---

Messrs. Fairchild Brothers & Foster, of New York, have placed us under obligations for a most useful desk diary for the year 1900, which this well-known firm has recently published. Besides a calendar and schedule of postage rates, it contains blank pages for memoranda for every day in the year upon each of which is printed, on the lower margin, some facts and general information relating to the digestive ferments and other preparations manufactured in the laboratories of this famed house. The book is printed on good paper, bound in strong muslin, and will be forwarded by the publishers upon application.

